# Early temporary ventricular assist device intervention improves survival in fulminant myocarditis with cardiogenic shock: experience from a single centre and national cohort

**DOI:** 10.3389/fcvm.2026.1727101

**Published:** 2026-02-23

**Authors:** Jiun-Yu Lin, Yi-Ting Tsai, Chih-Yuan Lin, Hung-Yen Ke, Jia-Lin Chen, Hsiang-Yu Yang, Wu-Chien Chien, Tsu-Hsuan Weng, Chien-Sung Tsai, Yi-Chang Lin, Po-Shun Hsu

**Affiliations:** 1 Division of Cardiovascular Surgery, Department of Surgery, Tri-Service General Hospital, National Defense Medical University, Taipei, Taiwan; 2 Department of Anesthesia, Tri-Service General Hospital, National Defense Medical University, Taipei, Taiwan; 3 Department of Medical Research, Tri-Service General Hospital, Taipei, Taiwan; 4 School of Public Health, National Defense Medical University, Taipei, Taiwan; 5 Graduate Institute of Life Sciences, National Defense Medical University, Taipei, Taiwan; 6 Medical Affairs Bureau, Ministry of National Defense, Taipei, Taiwan; 7 Department of Combat and Disaster Medicine, Tri-Service General Hospital, National Defense Medical University, Taipei, Taiwan

**Keywords:** acute fulminant myocarditis, cardiogenic shock (CS), ECMO—extracorporeal membrane oxygenation, intensive and critical care, VAD = ventricular access device

## Abstract

**Background:**

Acute fulminant myocarditis (AFM) complicated by cardiogenic shock (CS) often leads to rapid multi-organ failure. While extracorporeal life support (ECLS) is commonly used as an initial stabilizing measure, mortality remains high, particularly in patients requiring extracorporeal cardiopulmonary resuscitation (ECPR). Temporary ventricular assist devices (VADs) offer superior organ perfusion and more physiological hemodynamics compared to ECLS.

**Method:**

This retrospective study analyzed 16 AFM patients with CS who underwent VAD implantation following ECPR between December 2015 and February 2024. Clinical data, including laboratory profiles, vasopressor use, echocardiographic findings, and neurological status, were assessed. In parallel, survival outcomes from the Taiwan National Health Insurance Research Database (NHIRD) were compared among AFM patients treated with ECMO, VAD, or no mechanical circulatory support (MCS).

**Result:**

Of the 16 patients, 12 survived (75%) and 10 achieved cardiac recovery. Pre-VAD pulmonary artery pressure >40 mmHg and failure to wean from epinephrine by postoperative day 1 were associated with mortality. NHIRD analysis of 1,731 myocarditis patients showed the highest 30-day survival in the VAD group (88%) compared to ECMO (52%) or no MCS (71%; *p* = 0.003).

**Conclusion:**

AFM with CS often rapidly progresses to multi-organ failure. While ECMO provides initial circulatory support, survival remains poor once initiated. Early transition from ECMO to VAD is critical in improving survival for AFM patients with CS, particularly those receiving ECPR.

## Introduction

Acute fulminant myocarditis (AFM) is a rare but life-threatening disease marked by a cytokine storm and resulting immune dysregulation, leading to myocardial inflammation and impaired contractility (1). The resulting hyperinflammatory response may progress to cardiogenic shock, multi-organ failure, or death (2). Extracorporeal life support (ECLS) is commonly employed as initial mechanical circulatory support in critically ill AFM patients, though mortality remains high (28.7%–44%) (3). In contrast, short-term survival rates for AFM patients supported by ventricular assist devices (VAD) are significantly higher, ranging from 56% to 76% (4, 5).

End-organ dysfunction, particularly hepatic or renal impairment indicated by rising bilirubin, predicts poor prognosis and may warrant earlier VAD implantation to prevent irreversible damage (5). Multiple studies (6, 7) have shown that end-organ failure and myocardial injury are often irreversible, necessitating early VAD implantation to maintain circulatory support. VAD have made significant progress, improving organ perfusion and serving as a temporary solution for critically ill patients bridging to cardiac recovery or heart transplantation over the past two decades (8). VADs have evolved to provide effective left ventricular (LV) unloading and physiological flow, improving organ perfusion and bridging critically ill patients to either recovery or transplantation. Long-term data suggest that approximately 50% of patients survive post-VAD implantation through either myocardial recovery or successful transplantation (9).

This study aimed to assess the clinical outcomes of AFM patients with cardiogenic shock who received ECPR followed by temporary VAD support. Real-world evidence on the impact of VAD on mortality and long-term survival in this setting remains limited. We analyzed single-center data to compare survivors and non-survivors and used Taiwan's National Health Insurance Database to evaluate survival across patients receiving ECMO, VAD, or no mechanical circulatory support. These datasets were integrated to explore the potential survival benefit of VAD therapy in critically ill AFM patients.

## Material and methods

### Patient data sources and ethics

This study utilized both single-center data and the Taiwan National Health Insurance Research Database (NHIRD) to analyze outcomes in patients diagnosed with myocarditis. The single-center retrospective analysis included patients diagnosed with AFM at Tri-Service General Hospital between December 2015 and February 2024. All patients who developed cardiogenic shock (CS) underwent extracorporeal cardiopulmonary resuscitation (ECPR) and were subsequently transitioned to temporary VAD support due to clinical deterioration. Clinical parameters and survival outcomes were assessed. The institution's ethics committee approved the study protocol (TSGH-IRB number A202505082).

Additionally, survival outcomes were evaluated using NHIRD data from 2016 to 2020 for patients diagnosed with myocarditis. Due to the absence of detailed clinical information in the NHIRD, only survival outcome were analyzed in this cohort. The institution's ethics committee approved the study protocol (TSGH IRB No. E202516034).

### Study design, sampled participants and study outcomes

**Figure 1:**
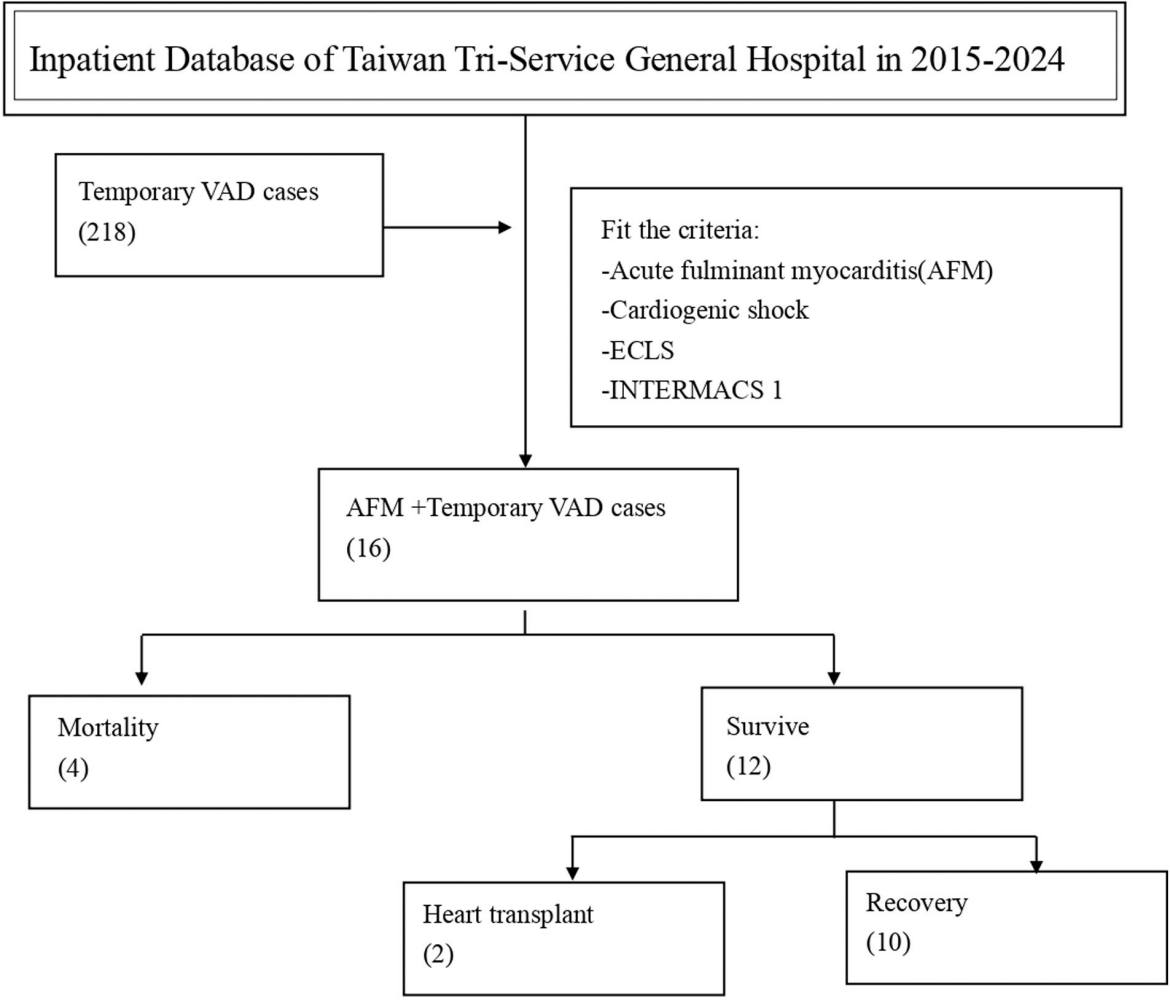
Patient selection flowchart from the inpatient database of Taiwan Tri-service general hospital (2015–2024).

The flowchart in Figure 1, demonstrate the inpatient database of Taiwan Tri-Service General Hospital between 2015 and 2024, a total of 218 cases undergoing temporary VAD implantation were identified. Among these, 16 patients met the specified inclusion criteria of AFM, CS, receiving ECLS, and categorized to Interagency Registry for Mechanically Assisted Circulatory Support (INTERMACS) profile 1. Patients who met the inclusion criteria were analyzed for in-hospital mortality and relevant clinical parameters.

In patients with acute AFM, CS is characterized by hypotension despite vasopressors, severe LV dysfunction (LVEF <30%), and signs of end-organ hypoperfusion. In cases progressing to cardiac arrest, CPR is initiated, followed by immediate ECMO support. The emergency ECLS set-up use Seldiger procedure usually through the femoral artery and vein.

AFM was diagnosed using an integrated clinical approach. All patients presented with acute severe heart failure or cardiogenic shock requiring inotropic support, E-CPR, or mechanical circulatory support. Emergent coronary angiography was performed in all cases to exclude obstructive coronary artery disease and other mechanical or structural causes of acute cardiac failure. Transthoracic echocardiography demonstrated severe ventricular systolic dysfunction (left ventricular ejection fraction <30%) in all patients.

Endomyocardial biopsy was performed when clinically feasible; however, histopathological examination did not consistently identify a specific infectious etiology or common myocarditis subtypes. Therefore, AFM was primarily diagnosed based on the clinical presentation, exclusion of ischemic and structural heart disease, and characteristic hemodynamic and echocardiographic findings.

#### Surgical technique and peri-operative management

Cannula size selection was individualized according to patient body size, body surface area, and anticipated target flow. For left ventricular support, a 32–34 Fr inflow cannula (Levitronix CentriMag, Waltham, MA, USA) was typically used via the right superior pulmonary vein, traversing the mitral valve into the left ventricle, while a 20–22 Fr outflow cannula was anastomosed to the ascending aorta. For right ventricular support, a 32 Fr inflow cannula was placed in the right atrium, and a 20–22 Fr outflow cannula was positioned in the main pulmonary artery. Ascending aortic and pulmonary artery outflow cannulation were performed using direct cannulation without side grafts in all cases.

Correct positioning of the left-sided inflow cannula was confirmed intraoperatively using transesophageal echocardiography (TEE), ensuring adequate placement across the mitral valve into the left ventricular cavity without suction events or ventricular wall contact. TEE was also used to assess ventricular unloading, septal position, and residual valvular regurgitation. discontinued ECLS and repaired the cannulated vessels with 6–0 Prolene sutures (10).

### Clinical data collection and analysis

In Table 1, baseline clinical, laboratory, hemodynamic, and echocardiographic variables were defined as those obtained during the cardiogenic shock phase prior to VAD implantation, while patients were supported with ECMO.

**Table 1:** Baseline clinical, laboratory, echocardiographic, and hemodynamic characteristics at shock phase prior to VAD implantation.

Variable	Overall (*N* = 16)	Survivors (*N* = 12)	Non-survivors (*N* = 4)	*p* value
Baseline characteristics				
Age (years)	36.9 ± 16.5	33.3 ± 16.0	48.0 ± 14.4	0.078
Female sex, *n* (%)	8 (50)	5 (41.7)	3 (75)	0.569
BMI (kg/m^2^)	26.2 ± 6.5	25.1 ± 6.6	29.7 ± 5.5	0.182
BSA (m^2^)	1.83 ± 0.3	1.79 ± 0.3	1.95 ± 0.3	0.225
Cardiopulmonary resuscitation relations				
CPR duration (min)	37.1 ± 47.0	36.1 ± 50.6	41.0 ± 36.5	0.557
ECMO–VAD interval (hours)	31.6 ± 43.1	36.2 ± 50.2	20.0 ± 15.9	0.888
Organs perfusion				
Brain injury, *n* (%)	5 (31.3)	3 (25)	2 (50)	0.547
Liver injury, *n* (%)	8 (50)	5 (41.7)	3 (75)	0.569
Renal dysfunction, *n* (%)	5 (31.3)	4 (33.3)	1 (25)	0.999
Arterial Blood Parameters				
pH	6.87 ± 1.8	7.34 ± 0.2	5.47 ± 3.6	0.302
Lactate (mmol/L)	4.38 ± 4.8	3.47 ± 0.83	4.09 ± 1.1	0.627
Hemodynamic characteristics				
MAP (mmHg)	79.3 ± 18.2	82.1 ± 18.1	70.8 ± 17.9	0.249
CVP (mmHg)	12.3 ± 10.6	10.9 ± 10.4	16.5 ± 11.5	0.262
HR(bpm)	117.56 ± 22.1	119 ± 24.3	113.25 ± 16.1	
Echocardiographic parameter				
LVEF (%)	16.5 ± 11.4	16.7 ± 13.1	15.6 ± 4.3	0.854
PAP (mmHg)	21.4 ± 16.7	15.1 ± 12.9	40.3 ± 12.7	0.009

BMI, body mass index; BSA, body surface area; CPR, cardiopulmonary resuscitation; CVP, central venous pressure; ECMO, extracorporeal membrane oxygenation; LVEF, left ventricular ejection fraction; MAP, mean arterial pressure; PAP, pulmonary artery pressure; VAD, ventricular assist device.

Baseline variables were obtained during the shock phase prior to VAD implantation. Liver injury was defined as AST or ALT ≥1,000 IU/L. Renal dysfunction was defined as the requirement for renal replacement therapy or elevated serum creatinine.

Statistics: Continuous variables are presented as mean ± standard deviation and categorical variables as number (%). Comparisons were performed using the Mann–Whitney *U* test or Fisher's exact test, as appropriate.

Relevant laboratory data were collected to assess myocardial injury and systemic perfusion, including arterial blood gas analysis, lactate levels, renal and liver function tests. Hemodynamic parameters such as mean arterial pressure (MAP), central venous pressure (CVP), and heart rate (HR) were recorded. Transthoracic echocardiography was performed to evaluate left ventricular ejection fraction (LVEF), pulmonary artery pressure (PAP), left ventricular end-systolic diameter (LVESD), and left ventricular end-diastolic diameter (LVEDD). The use of inotropic and vasopressor agents was documented as part of perioperative clinical assessment. The duration of CPR was defined as the cumulative time on cardiac massage, excluding periods of return of spontaneous circulation, the interval to VAD was defined as the time from the initiation of ECLS to the documented establishment of VAD. Shock-induced liver failure, defined as AST and ALT levels rise to 1000 IU/L or more (11).

**Figure 2:**
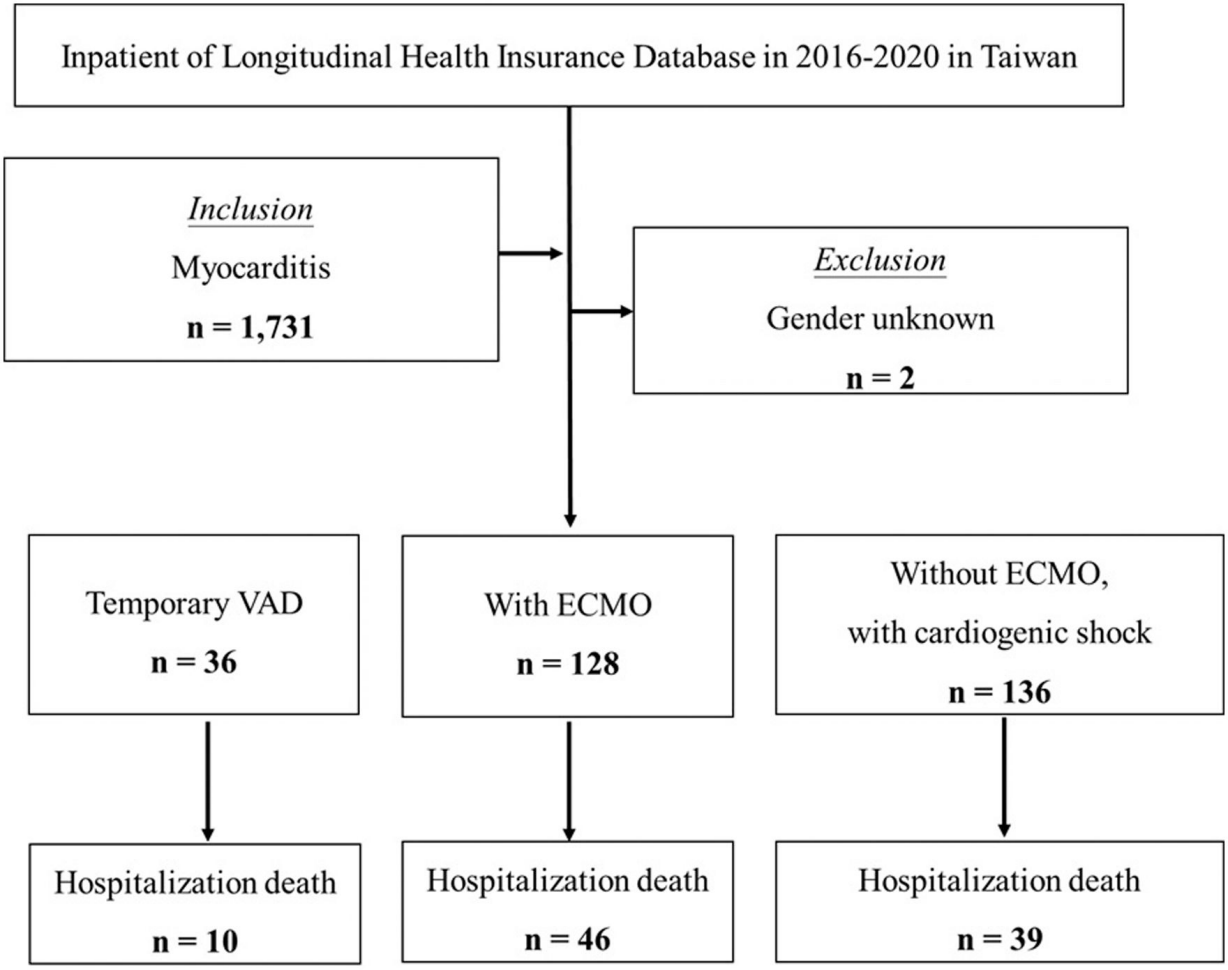
Patient selection flowchart from NHIRD (2016–2020).

In Figure 2, patient selection flowchart from the Longitudinal Health Insurance Database (2016–2020, Taiwan). The Levitronix CentriMag (Waltham, MA, USA) VAD was not reimbursed by Taiwan's National Health Insurance until December 2015. A total of 1,731 patients diagnosed with myocarditis were identified. After excluding 2 cases with unknown gender, patients were categorized into three groups: those who received temporary VAD support (*n* = 36), those treated with ECMO (*n* = 128), and those who had CS but did not receive ECMO (*n* = 136). The number of in-hospital deaths in each group was 10, 46, and 39, respectively. Given the lack of clinical data within the NHIRD, analysis for this cohort was limited to survival outcomes.

### Statistical analysis

SPSS 25.0 statistical software (SPSS Inc., Chicago, IL, USA) was used for all analyses, and a *p*-value less than 0.05 was considered statistically significant. Continuous variables were expressed as means ± standard deviation and were compared using the unpaired t. Multivariate Cox proportional hazard regression was used to determine the risk for mortality, and the results were presented as hazard ratio (HR) with 95% confidence interval (CI). The difference in the risk for mortality was estimated using the Kaplan–Meier method with the log-rank test.

## Results

Baseline characteristics and clinical severity as shown in Table 1, a total of 16 patients with acute fulminant myocarditis complicated by cardiogenic shock were included, of whom 12 survived and 4 died during hospitalization. Baseline variables were collected during the shock phase prior to VAD implantation.

Non-survivors tended to be older than survivors (48.0 ± 14.4 vs. 33.3 ± 16.0 years), although this difference did not reach statistical significance (*p* = 0.078). There were no significant differences between the two groups with regard to sex, body mass index, body surface area, or pre-existing cardiac comorbidities, including diabetes mellitus, hypertension, hyperlipidemia, coronary artery disease, or dilated cardiomyopathy.

The duration of cardiopulmonary resuscitation and the interval from ECMO initiation to VAD implantation were comparable between survivors and non-survivors. The incidence of CPR-related organ injury, including brain, liver, and renal dysfunction, did not differ significantly between groups.

With respect to baseline laboratory and hemodynamic parameters, non-survivors demonstrated a trend toward more severe metabolic derangement, including lower pH and higher lactate levels; however, these differences were not statistically significant. Baseline mean arterial pressure and central venous pressure were similar between groups.

Importantly, baseline echocardiography during shock revealed significantly higher pulmonary artery pressure (PAP) in non-survivors compared with survivors (40.3 ± 12.7 vs. 15.1 ± 12.9 mmHg, *p* = 0.009), whereas left ventricular ejection fraction and ventricular dimensions did not differ significantly.

Perioperative hemodynamics, vasopressor use, and laboratory trends present in Table 2. On postoperative day 1, epinephrine use was significantly more frequent among non-survivors than survivors (75.0% vs. 8.3%, *p* = 0.027). In contrast, the use of dopamine, dobutamine, and norepinephrine did not differ significantly between groups at any time point. Overall, a decreasing trend in vasopressor requirements was observed during the first three postoperative days, with the most pronounced reduction noted in epinephrine use.

**Table 2:** Perioperative hemodynamics, vasopressor use, and laboratory parameters after VAD implantation.

Variable	Survivors (*N* = 12)	Non-survivors (*N* = 4)	*p* value
Inotropic and Vasopressors use on POD1			
Epinephrine, *n* (%)	1 (8.3)	3 (75)	0.027
Dopamine, *n* (%)	5 (43.8)	2 (50)	0.999
Norepinephrine, *n* (%)	6 (50)	2 (50)	0.999
Arterial Blood Parameters on POD1			
PH	7.46 ± 0.1	7.42 ± 0.1	0.274
Lactate (mmol/L)	2.72 ± 1.2	4.24 ± 1.3	0.182
Hemodynamic characteristics on POD1			
MAP (mmHg)	88.7 ± 15.5	87.8 ± 10.3	0.999
CVP (mmHg)	16.4 ± 4.1	23.0 ± 8.3	0.127
Heart Rate(BPM)	102.75 ± 15.3	91.25 ± 24	0.585

Postoperative values were recorded on postoperative day 1 following VAD implantation.

Statistics: Continuous variables are expressed as mean ± standard deviation and categorical variables as number (%). Between-group comparisons were performed using the Mann–Whitney *U* test or Fisher's exact test.

CVP, central venous pressure; MAP, mean arterial pressure; POD, postoperative day.

Hemodynamic parameters, including mean arterial pressure, central venous pressure, and heart rate, showed no statistically significant differences between survivors and non-survivors. Nevertheless, postoperative trends demonstrated improved mean arterial pressure and stabilized heart rate, particularly in the survivor group.

Regarding arterial blood gas and laboratory parameters, non-survivors exhibited a trend toward more severe preoperative acidosis and higher lactate levels, suggesting a greater burden of end-organ hypoperfusion. However, postoperative improvements in acid–base status and lactate clearance were observed in both groups, and no statistically significant intergroup differences were identified.

Echocardiographic findings and clinical outcomes are presented in Table 3. During the shock phase, pulmonary artery pressure was significantly elevated in non-survivors, whereas other echocardiographic parameters, including left ventricular ejection fraction and ventricular dimensions, were comparable between groups. Prior to VAD removal, echocardiographic parameters showed no significant differences, indicating partial hemodynamic stabilization among patients who survived to this stage.

**Table 3:** Clinical course and Survivors long-term outcomes.

Variable	Survivors (*N* = 12)	Non-survivors (*N* = 4)	*p* value
PAP during shock (mmHg)	15.1 ± 12.9	40.3 ± 12.7	0.009
VAD durations (Hours)	350.2 ± 19.1	266.2 ± 19.2	0.808
ICU days	45.6 ± 15.9	9.5 ± 2.6	0.145
Hospital days	66.1 ± 18.8	9.5 ± 2.6	0.181
Survivors long-term outcome (*N* = 12)			
	Cardiac recovery, *n* (%)	Heart transplantation, *n* (%)	
	10 (83.3)	2 (16.7)	

ICU, intensive care unit; PAP, pulmonary artery pressure; VAD, ventricular assist device.

Notes: Echocardiographic parameters during shock were measured prior to VAD implantation.

Statistics: Continuous variables are presented as mean ± standard deviation and categorical variables as number (%). Comparisons were conducted using the Mann–Whitney *U* test or Fisher’s exact test.

Among the 12 survivors, 10 patients (83.3%) achieved native cardiac recovery, while 2 patients (16.7%) underwent heart transplantation (*p* = 0.008). Support duration metrics, including oxygenator duration, VAD support duration, ventilator days after VAD implantation, and total ICU or hospital length of stay, did not differ significantly between survivors and non-survivors.

Echocardiographic findings and clinical outcomes are presented in Table 3. During the shock phase, pulmonary artery pressure was significantly elevated in non-survivors, whereas other echocardiographic parameters, including left ventricular ejection fraction and ventricular dimensions, were comparable between groups. Prior to VAD removal, echocardiographic parameters showed no detectable differences, indicating partial hemodynamic stabilization among patients who survived to this stage.

Among the 12 survivors, 10 patients (83.3%) achieved native cardiac recovery, while 2 patients (16.7%) underwent heart transplantation (*p* = 0.008). Support duration metrics, including oxygenator duration, VAD support duration, ventilator days after VAD implantation, and total ICU or hospital length of stay, did not differ detectable difference between survivors and non-survivors.

**Figure 3:**
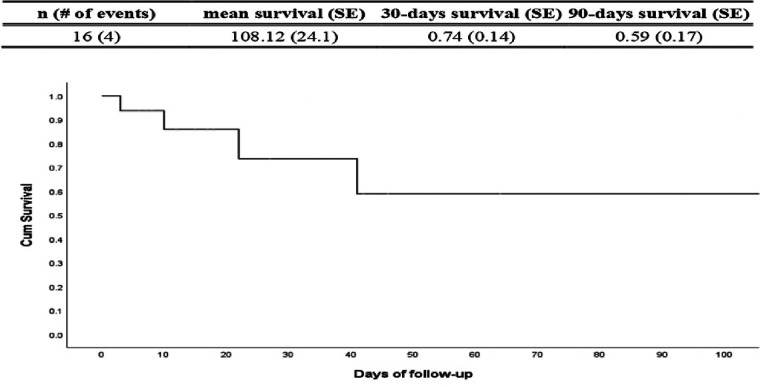
Kaplan-Meier analysis all-caused accumulative survival. Declaration of generative AI and AI-assisted technologies in the writing process. During the preparation of this work the authors used Grammarly and OpenAI in order to improve readability. After using this service, the authors reviewed and edited the content as needed take full responsibility for the content of the publication.

Figure 3 is a Kaplan–Meier analysis showed all-cause accumulative survival, among the 16 study subjects, 4 experienced mortality. The average survival time of each individual was 108.12 days. The survival rate of 30 days was 74%, and 59% after 90 days.

**Figure 4:**
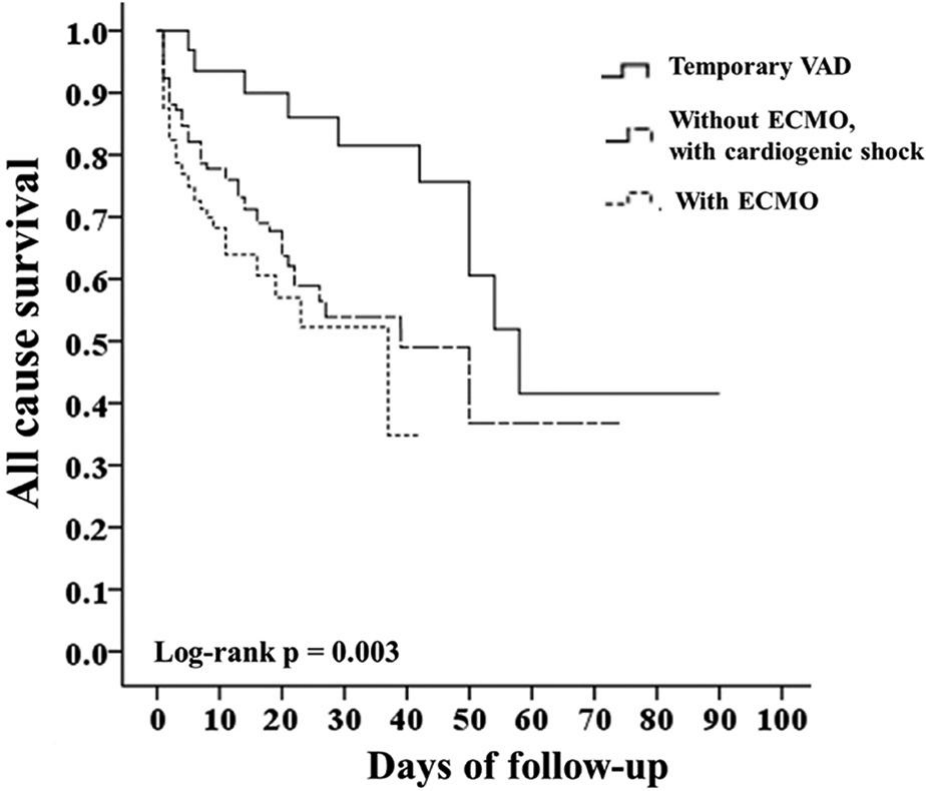
Kaplan-Meier survival curve- with, without ECMO and VAD.

In Figure 4, Taiwan NHIRD from 2016 to 2020, focusing on patients diagnosed with myocarditis. A Kaplan–Meier survival analysis comparing three patient groups—Temporary VAD, without ECMO, and ECMO alone—demonstrated distinct prognostic differences. Temporary VAD had highest survival rates over the 100-day follow-up period, patients who did not require ECMO exhibited the intermediate survival rates, the ECMO group had the lowest survival rates, with the steepest decline observed early in the course of treatment. Notably, the temporary VAD intervention significantly improved survival compared to ECMO alone. The difference in survival among the three groups was statistically significant (log-rank *p* = 0.003).

### Predictors of postoperative mortality in TSGH VAD-support

Mortality group had significantly higher PAP during cardiogenic shock (40.25 vs. 15.08 mmHg, *p* = 0.009) and a greater need for epinephrine on postoperative day 1 (75% vs. 8.3%, *p* = 0.027). These findings suggest that persistent pulmonary hypertension and vasopressor dependence are strong predictors of mortality in AFM patients escalated to VAD.

### VAD survival rate of Taiwan NHIRD dataset

The NHIRD data indicate that AFM patients with CS, temporary VAD support confers a clear survival advantage over ECMO alone, with non-ECMO shock patients showing intermediate outcomes.

## Discussion

### Impact of elevated PAP and inotropic dependence on outcomes

Persistently elevated pulmonary artery pressure (PAP) during the cardiogenic shock phase prior to VAD implantation was strongly associated with mortality in our cohort. Non-survivors demonstrated significantly higher baseline PAP compared with survivors (40.3 ± 12.7 vs. 15.1 ± 12.9 mmHg, *p* = 0.009), suggesting more advanced biventricular failure at presentation. Importantly, these measurements were obtained under ECMO support before VAD implantation, emphasizing PAP as a baseline hemodynamic severity marker rather than a post-intervention finding.

Persistently elevated pulmonary artery pressure (PAP > 40 mmHg) after ECMO initiation is associated with increased mortality. Studies suggest that VA-ECMO does not sufficiently unload the RV and may increase LV afterload due to retrograde aortic flow (12), thereby elevating pulmonary pressures and exacerbating RV strain. Patients requiring ECMO with concurrent vasopressor use or metabolic acidosis exhibit significantly higher in-hospital mortality, indicating that sustained PAP elevation may reflect irreversible cardiac dysfunction (13). Therefore, ventricular unloading may be a critical determinant of survival in AFM patients receiving ECMO support.

In contrast to baseline PAP, continued epinephrine requirement on postoperative day 1 represents an early physiological response after VAD implantation. In our study, non-survivors were significantly more likely to require epinephrine on postoperative day 1 (75% vs. 8.3%, *p* = 0.027), suggesting persistent circulatory insufficiency despite full mechanical support. This finding aligns with previous reports indicating that early vasopressor dependence after VAD implantation is associated with poor myocardial reserve, right ventricular failure, and increased mortality.

Persistent pulmonary hypertension may have limited effective RV unloading, resulting in reduced LV preload, low cardiac output, and ongoing vasopressor dependence. In addition, systemic inflammatory vasoplegia following prolonged cardiogenic shock and ECPR may have contributed to reduced vascular tone, further necessitating catecholamine support. High vasoactive-inotropic score (VIS) after L-VAD surgery, especially involving high doses of epinephrine, is significantly associated with in-hospital mortality (14). High-dose epinephrine contributes to elevated myocardial oxygen demand and is linked to early right ventricular failure, a major cause of post-VAD mortality (15–17). This aligns with prior evidence that early weaning from vasopressors correlates with improved outcomes in cardiogenic shock patients supported with ECMO or VAD.

Inhaled nitric oxide (iNO) is a selective pulmonary vasodilator used in acute right ventricular failure and pulmonary hypertension. By reducing pulmonary vascular resistance, iNO lowers RV afterload and improves RV output without causing systemic hypotension (18). Although there are a few case reports indicating that iNO for LVAD or ECMO serves as an important adjunctive therapy to stabilize pulmonary hemodynamics and support right heart recovery (19). However, large-scale randomized clinical trials, such as that by Potapov et al. (2011) (20), found that while iNO improved short-term hemodynamic stability after LVAD implantation, it did not significantly reduce the incidence of right ventricular failure, the need for RVAD, or overall mortality.

Although current evidence does not demonstrate a significant reduction in mortality, iNO remains a valuable adjunctive therapy that merits further investigation as part of an integrated hemodynamic optimization strategy in advanced mechanical circulatory support.

### VAD rather than ECMO provides normal physiological blood flow direction and LV unloading

VA-ECMO may inadequately unload the LV and increase afterload due to retrograde aortic flow, thereby exacerbating pulmonary congestion and right ventricular strain. In this context, elevated baseline PAP during shock may reflect the cumulative effects of severe left ventricular dysfunction, pulmonary venous hypertension, and secondary right ventricular failure. This hemodynamic profile is consistent with prior studies demonstrating worse outcomes in cardiogenic shock patients with persistent pulmonary hypertension despite ECMO support.

Increased LV afterload can result in increased PAP and risk of pulmonary edema and a subsequent deterioration in oxygenation, myocardial ischemia, delayed ventricular recovery, ventricular arrhythmias, thrombotic events, and multiorgan dysfunction in CS status (21). The mortality rate for patients with AFM on VA-ECMO is approximately 40% (3). Besides, the flow is constrained by the narrow caliber of the peripheral artery, which adversely affects the perfusion of the cannulated limb. Asian individuals appear to experience this phenomenon more severely, possibly due to smaller body size and femoral artery diameter (22). The CentriMag VAD device has been considered a bridge-to-decision therapy, serving as a temporary VAD for the management CS. VAD can ensure adequate circulatory support for the organ and unload the LV with direct cannulation of the VAD cannula to the atrium and major vessels, ensuring antegrade flow to prevent any limitations in circulation. Consequently, the Bi-VAD configuration effectively mitigate the workload of both ventricles concurrently and independently.

Other less invasive methods for LV unloading commonly used methods include: ECPella (ECMO + Impella), IABP + ECMO, and transseptal ventilation (through atrial septostomy and left atrial drainage). These strategies aim to reduce left ventricular afterload, improve cardiac output, and prevent complications such as LV dilatation and pulmonary edema (23, 24). The mortality reduction was consistent across studies, with risk ratios ranging from 0.79 to 0.89. LV unloading was also associated with improved ECMO weaning success and better neurological outcomes (25). While ECPELLA has shown superior LV unloading, some studies found no significant difference in in-hospital mortality between ECPELLA and ECMO + IABP (26). However, unlike BiVAD, these methods do not support pulmonary circulation and are insufficient for maintaining long-term physiological perfusion in severely damaged hearts and the risks such as aortic valve injury (27–29) and fatal cardiac perforation (30) may occurred.

### Early BiVAD escalation can improve survival and cardiac recovery in AFM

The survival rate for patients with AFM treated with ECMO alone, a meta-analysis reported survival rate of approximately 66.9% for adult patients (3). The survival rate of combination ECMO and VAD was 63% survived to discharge, with better outcomes observed at transplant centers (83% survival) compared to non-transplant centers (55% survival) (4, 31). Our study enrolled the extremely critical patients (“Crash and burn” INTERMACS profile 1) and all cases experienced CPR for average 37 min. The statistical findings indicate that the recovery rate for patients shift to VAD following emergency ECMO use was 63% (10 out of 16). To include two who survived post-transplantation, resulting in an overall survival rate of 75% (12 out of 16). Compared to other reports, our patients were among the most critically ill, yet showed equal or better survival rates—highlighting the importance of early VAD intervention.

In Figure 3, Kalan-Meier analysis all-caused accumulative survival analysis of data demonstrates that AFM patients experiencing CS who are escalated to VAD therapy achieve a 30-day survival rate of up to 74%, significantly higher than those receiving ECMO alone (32). Patients with myocarditis complicated by cardiogenic shock who require ECMO have a significantly lower 30-day survival rate (33). This suggests that when myocarditis patients progress to a stage requiring ECMO, their condition may have reached an irreversible level of cardiac dysfunction. Consequently, ECMO can only provide temporary bridging support and is unlikely to serve as a definitive treatment option. Clinically, assessment of patients' cardiac recovery potential and consideration for early escalation to long-term VAD support is essential to improve survival outcomes. These findings highlight the critical role of VAD as a therapeutic strategy for sever e heart failure associated with myocarditis, emphasizing that earlier intervention with VAD in patients already supported by ECMO correlates with higher survival and better cardiac recovery (4, 5).

### Efficacy of ECMO and VAD for AFM with cardiogenic shock in NHIRD

We analyzed data from the Taiwan NHIRD from 2016 to 2020, focusing on patients diagnosed with myocarditis. Figure 4 is a Kaplan–Meier survival analysis comparing three patient groups—Temporary VAD, without ECMO, and ECMO alone—demonstrated distinct prognostic differences. Temporary VAD had highest survival rates, patients who did not require ECMO exhibited the intermediate survival rates, the ECMO group had the lowest survival rates, with the steepest decline observed early in the course of treatment. Notably, the temporary VAD intervention significantly improved survival compared to ECMO alone (*p* = 0.003). Our another single center cohort study show temporary VAD should be applied if ECLS could not rescue the sustained CS with promising outcome (10).

Although the nationwide NHIRD analysis demonstrated a survival advantage associated with temporary VAD support, these findings should be interpreted with caution. The observed differences may be influenced by confounding by indication, as patients selected for VAD therapy likely survived long enough to recieved VAD implantation. Immortal-time bias cannot be excluded.

Owing to the absence of precise timing of interventions in the NHIRD, the national-level survival comparisons should be regarded as hypothesis-generating rather than definitive evidence of causal benefit. It should also be acknowledged that the adoption of temporary VAD therapy is highly dependent on institutional volume, available expertise, and referral pathways, which may have influenced patient selection and treatment allocation in both the single-center cohort and the nationwide database analysis.

### Limitations

First, this was a retrospective study, rely on existing medical records, which may contain inconsistencies or missing data which is prone to selection bias during data collection. As mentioned, VAD was introduced at our center after 2015. A prospective randomized controlled trial would therefore be essential to generate more robust evidence. Second, the sample size in this study was relatively small (*n* = 16). However, recruiting AFM cases is inherently challenging, particularly among patients requiring ECPR and escalating VAD support. In such an extremely critical population, substantial variability across clinical variables is expected. Prospective cohort studies or propensity-matched analyses could help reduce bias and enhance the validity of future findings.

Finally, the NHIRD provides data based on diagnostic and procedural codes, limiting the ability to determine the temporal sequence of interventions or assess disease severity. Furthermore, granular clinical parameters—such as hemodynamic status, laboratory values, or response to treatment—are not available. Despite these limitations, the large sample size and nationwide coverage of the database allow for meaningful population-level analysis and can offer valuable insights for clinical and epidemiological research.

To address these limitations, future research should consider as conducting multi-center studies to increase the external validity of findings. Expanding sample sizes to improve statistical power and enable subgroup analyses.

## Conclusions

AFM is a severe, life-threatening condition characterized by rapid cardiac deterioration, often leading to cardiogenic shock and multi-organ failure. In cases of AFM with cardiogenic shock, ECMO is the first-line intervention to provide circulatory and respiratory support, protecting organs such as the brain, kidneys, and liver from ischemic injury. However, once ECMO is initiated in such critical patients, the survival rate remains extremely low. Early escalation to VAD support for unloading LV and secure pulmonary circulation are essential to improve outcomes.

## Data Availability

The original contributions presented in the study are included in the article/Supplementary Material, further inquiries can be directed to the corresponding authors.
